# Cholera Viewed from a New Standpoint

**Published:** 1876-04

**Authors:** Henry R. Rogers

**Affiliations:** Dunkirk, N. Y.


### EXAMINER.

Vol. XXXIII. — APRIL, 1876. — No. 4.

### Original Communications.

### CHOLERA VIEWED FROM A NEW STANDPOINT.

A paper read before the Chautauqua County Medical Society, January 18th, 1875, introductory to discussion,

By HENRY R. ROGERS, M.D., Dunkirk, N. Y.

This disease, from its peculiar character and fatal effects, has engaged the attention of investigators and writers to an extent scarcely equaled by any other in the catalogue of maladies.

The time, the careful study, the patient investigations, and the apparently exhaustive discussions which have been devoted to the elucidation of this great and vitally important subject, ought to have secured to us something more tangible in positive knowledge of its nature, its cause, its dissemination and its treatment.

The opinions which prevail to-day prevailed more than a quarter of a century ago, and the treatment of to-day scarcely differs from that employed for many years past. The whole pharmacopœia has been drawn upon for remedies with which to combat it, and, *of the innumer-*

*able forms of treatment made use of, there is scarcely any variation in the results.*

Have investigators never found themselves querying, whether the views upon the subject which have been held as indisputable, might not, after all, prove to be erroneous?

Whatever differences of opinion may have obtained regarding minor points, in one or two respects there has been remarkable unanimity. The opinion that it is an idiopathic disease of the primæ viæ, or of the blood and primæ viæ, has passed almost unquestioned, from its earliest history to the present day. From this central standpoint have radiated the thousand and one ideas as to its nature, cause and treatment, and nearly all studies, all investigations, and all arguments, have been founded upon this axiomatic basis.

I may be permitted, and, too, I hope without the charge of egotism, to assume a different standpoint.

Some of the ideas advanced will not be those usually entertained by the profession, yet I hope they may be received without prejudice, entertained with candor, and judged dispassionately. They combine the results of personal experience and observation both in this country and abroad, during the various epidemics which have occurred in the past twenty-five years, also the results of an experience of the writer derived from an attack of the disease.

I. *Nature.*—The cholera I regard to be essentially *a disturbance of innervation.**

Its various phenomena may be more satisfactorily accounted for upon this, than upon any other hypothesis. The following are a few of the grounds upon which I found my conclusions:

1. The *purging*, or, *more properly speaking*, the *transudations*, in the profuseness and painlessness

* "Innervation. The nervous influence necessary to the maintenance of life and the functions of the various organs." DUNGLISON.

which characterize them, *could not occur*, *except the nerves*, *whose function it is to preside over the parts implicated, fail to perform their duties in a normal manner.*

The unlocking of the exhalent orifices of the blood vessels, *permitting* the rapid filtration into the stomach and intestinal canal, of the finer elements of the blood, and sometimes of the blood itself, *is a positive evidence of perturbation of tone in the membrane through which the infiltration takes place.*

This abnormal condition cannot be claimed to result from the presence of inflammation, *neither does any process of poisoning, by any substance known to man, produce a similar effect upon the nervous system.*

The excessive sweating arises from the same general cause.

2. The *cramps* arise from purely nervous causes.

3. The *vomiting* is simply regurgitative.

4. The *collapse* is usually due to the rapid withdrawal of the natural stimulus of the brain, *i. e.*, healthy blood, in normal quantities, yet it also arises from the initial force of the invasion of the disease.

5. In the more rapidly fatal cases in severe epidemics, death sometimes results from the initial force of the disease *expending itself upon the brain and nervous system*, and it takes place even before the serous elements of the blood have entered the primæ viæ.

6. No pathological changes are discoverable when death has resulted at an early period of the disease, *neither have any constant and uniform pathological conditions been observed after death*, *occurring in any of its stages.*

The epithelial changes are unimportant, *as they never produce appreciably unfavorable effects.*

7. When the disease is arrested in its earlier stages, and, too, not unfrequently in cases where the symptoms have been most grave, the recovery is immediate, and no effects upon the system are observable, other than those resulting from the loss in the quantity of the circulating fluid.

8. The cause most favorable to its production during the prevalence of epidemics is *fear*. This, the most powerful of all nerve depressors, directly favors, and also invites attack.

II. *Cause.*—The theory of "*a peculiar epidemic influence as an essential and specific cause*," offers a solution of the problem, which, if not entirely satisfactory, is open to as few objections as any other.

The facts, that the disease in its migrations overleaps immense distances, where no human agencies could account for it; that it travels against winds; and that it prevails in temperatures below zero, should receive more consideration than is usually awarded them, in estimating its causes. The first is opposed to the theory of a specific poison, and theg enerally accepted germ theory is inconsistent with the two latter.

III. *Dissemination.*—Other influences contribute in spreading the disease when once originated. What these influences are, and in what manner they produce their results, yet remains a mystery. The commonly accepted proposition, that it travels by routes which may be traced on maps with lines made with almost mathematical accuracy, cannot be sustained by facts. The data upon which this conclusion is founded, are too unreliable to admit of positive theories being founded upon them.

That the influence during prevailing epidemics is more generally pervasive than has been heretofore supposed, I fully believe. It is received or not into the various systems, according to their different degrees of susceptibility. Persons thoroughly impregnated may escape an active attack, if no exciting cause be present to awaken it into action, and the subject later becomes freed from its presence by spontaneous elimination.

IV. *Treatment.*—The treatment of this disease, if the foregoing premises are correct, emerges from empiricism and becomes thoroughly scientific. It becomes also simplified to the last degree. The remedies selected should be prompt in their effects in correcting disturbed innervation ; even moments of delay may sometimes result in the sacrifice of a life.

Influenced by my appreciation of the character of the disease, and impelled by the urgency of my cases, I have in my latest experience made use of the acetate of morphia, hypodermically applied, in its treatment. This produces less of dizziness and nausea than the sulphate or hydro-chlorate.

It acts quickly and efficiently.

*No one may know, without a personal experience, the value of this agent, thus administered, in controlling this disease.*

The main indication in the treatment being thus met, other conditions incident to the disease may properly be treated upon general principles.

The *brain and heart* becoming enfeebled by the rapid abstraction of the serous elements of the blood, the same condition is produced as that resulting from the rapid loss of a large quantity of blood, (as in the flooding of parturition or abortion,) for which the horizontal position is necessary, and, if the discharges be profuse, the safety of the patient renders it *imperative* that the head be kept lower than the body, for a considerable period of time, that those organs may become in a measure resupplied by gravitation.

The *stomach* becomes morbidly sensitive. Its demands for liquids, in consequence of the rapid abstraction of the fluids of the body, become excessive, and the thirst intense, at the same time the mucous membrane will not tolerate the presence of water, much less of food or medicines. This condition is an indication for the internal employment of ice ; this cools the burning heat of the stomach and in a degree allays the thirst, and the water is so rapidly absorbed that it does not become a source of irritation and provoke vomiting.

The *bowels*, as sensitive as the stomach, require something to diminish the sensibility along their whole track, and to shut up the millions of patulous orifices from which the stream of life is flowing. This indication is met by the remedy proposed.

The *nerves* in their abnormal condition throughout the system require immediate control to give relief from sufferings. This is also met by the same remedy.

The *circulation*, necessarily depressed, should be promoted by external heat, frictions, etc.

The *collapse* should be treated by position, external heat, and stimuli, also by enemas containing nutriment and stimuli. Opiates are harmful in this stage.

En résumé, the treatment resolves itself into—

1. The hypodermic use of acetate of morphia, administered according to the age and condition of the patient. In cases of no great urgency, other remedies and methods of administration may be employed. Opium and its preparations are preferable, however administered.

2. The horizontal position, with the head as low as the body or lower.

3. *For the mouth, nothing but finely pounded ice, and that ad libitum.* Where this cannot be obtained, the coldest water may be given and frequently repeated in smallest quantities, until it can be tolerated without provoking vomiting.

4. External heat, frictions, etc.

5. For collapse: position, external heat and stimuli, and, by enemas, nutriment and stimuli.

6. After-treatment for restoring wasted forces.

A few cases in illustration of this treatment, may serve in a measure to give a practical character to this paper. Of the cases treated by the writer (eleven in number), eight were grave and typical. In all, *the pathognomonic symptom* (the rice-water discharges) was present.

As in all of them the train of symptoms and the treatment were about the same as already suggested, it will not be important to fully describe either, in symptoms or treatment. I will, therefore, mention such points as may be deemed of interest.

1. Mr. O'D., aged about 50 years. After a diarrhœa of thirty-six hours continuance in comparatively mild form, the symptoms became more grave; vomiting, profuse rice-water discharges, cramps of the abdomen and legs, blue and shriveled skin and husky voice, were present when I was called in attendance. I found the pulse feeble, and numbering 140 per minute, and the prostration was extreme. My diagnosis was cholera, and the prognosis, fatal. The treatment was nearly as described; the acet. morph. was hypodermically administered, in a quarter of a grain dose, and acted favorably in relieving the most urgent symptoms, but it was not repeated, as collapse was soon present, and the patient sank gradually, expiring a few hours after my first visit. In this case stimulants and usual cholera remedies were employed, in addition to those mentioned.

2. Mr. M., aged about 55 years; health fair, but in person emaciated; habits regular, and no assignable cause for the attack. After a slight diarrhœa of some hours continuance, the invasion was abrupt, with usual symptoms in high degree. Morphia, position, ice, frictions, and external heat, were employed. No other medicaments were used. The result was favorable.

3. Was a repetition of the above.

4. Mrs. F., a German woman of 45 years, a resident of the country. I saw her first after many hours of illness; the vomiting, purging and cramps were most distressing and very frequent, and her condition was one of greatest prostration. The case was very unpromising, and the family had quite despaired of her recovery. The disease responded promptly and favorably to the treatment; the recovery was slow, but otherwise satisfactory; no other medicines were administered.

5. Miss F., aged about 30 years, a resident of the country; had been ill many hours when I was called in attendance, and her condition on my arrival was alarming; the pulse was rapid and feeble, and the forces well nigh exhausted by reason of previous discharges and sufferings. On entering the sick room I at once applied the acet. morphiæ in dose of quarter of a grain, hypodermically, and almost immediately afterwards she was again seized with cramps; for a short time frictions, heat, etc., were employed without apparent benefit, she then begged to be permitted to stand upon her feet, as that position had previously given her relief when other means had failed. I could not resist her appeals, emphasized as they were by her sufferings, and with a mental protest I gave the desired permission and retired from the room. I was soon recalled by her sisters, who said that she was dead. On returning, I found her pulseless and pallid; after a time slight reaction took place, and under the circumstances, I regarded it my duty to administer stimulants; these were quickly ejected and appeared rather to increase her sufferings; the treatment from thence was solely by hypodermic injections and the accompaniments mentioned. In this case, as in nearly all of the others, the applications of morphia were made at intervals of from three to six hours, and in doses of one-sixth to one-quarter of a grain. The result was favorable, yet the recovery was slow.

6. Mr. F., aged about 40 years, a night watchman in a machine shop; health for several years impaired; on Saturday evening he was in his usual health, and, before commencing his labors, partook of his supper with a fair appetite; at 11 o'clock he was attacked with a diarrhœa, which was soon attended with vomiting and cramps; he persisted in remaining at his post of duty until 5 o'clock, A. M., at which time he was able only by extraordinary exertions to reach his home, but a few blocks distant. Presenting himself at his door for admission, his wife did not recognize his voice, now so husky, and on entering, she was as much surprised at the change in his countenance as before in his voice. His features were shrunken, his gait tottering, extremities cold, the cramps severe, and the thirst intense. I was summoned at once, and found the pulse rapid and feeble, and the prostration extreme. The evacuations, of which there were two within half an hour after my arrival, amounted to nearly two quarts, and were the purest rice-water in appearance. His condition was such that with my experience in using morphia I dared not apply it. I demanded council, as I wished to make use of a means unusual. I have for the past twenty-five years entertained my present opinion regarding the nature of this disease, and have been desirous of using chloroform to meet some of the indications for which I now use the acet. morphia. This case warranted us in employing any means that offered the slightest hope of benefit. Before commencing the use of the chloroform, the tendency to collapse was so great that I regarded it as my first duty to stimulate the brain and heart to increased action, by sending to those organs a current of blood by gravitation. The head was quickly placed several inches lower than the body. I then commenced to administer chloroform by inhalation and in five minutes he was soundly sleeping ; at the end of ten minutes more he was awakened by cramps, and I soon renewed the chloroform and gave him half an hour more of sleep. Nearly an hour had now elapsed since depressing the head, and, on examining the pulse, I found that it had slightly more of firmness, which encouraged me to commence the use of the morphia ; this I administered in usual doses, and relied upon it during the remainder of the treatment.

The patient not being responsible for his actions, a strong man was placed in charge of him to preserve his position ; this he was inclined to resist for many hours. He recovered slowly but satisfactorily.

7. This case in its features and treatment resembled case 3, and the results were the same.

8. Mrs. G., young, and in good health, and with habits of diet and living as perfect as may be, was, on the 7th of November, as free as possible from any indications of the presence of this disease, until about 7 P. M., at which time she was actively attacked, and the symptoms rapidly increased in severity. At 10 o'clock, when standing in her parlor, she dropped helplessly upon the floor. I arrived a short time subsequently, and found her almost in a state of collapse. The pulse was rapid and slightly perceptible, the eyes were sunken and expressionless, the countenance pinched, the skin of a purplish shade, and the voice husky. The mother, who was present, remarked that there was scarcely a feature by which she could recognize her as her daughter.

The acet. morph. was applied, and decided relief was obtained within twenty minutes, and in one hour all the untoward symptoms were under control.

9, 10, and 11. In these, the characteristic symptoms were present, but the cases were not so severe as the preceding. The treatment and results were the same.

Besides the cases of cholera described, I was called upon to treat four cases of cholerine and many cases of cholera-morbus, during the season. The remedial and other treatment were the same, and the results most gratifying.

In the cases cited, the results were very favorable, and although few in number, the gravity which characterized them would militate against the idea of simple coincidences.

In hospital practice, or in a prevailing epidemic, no such favorable results could reasonably be expected. The faithful nursing and careful watching which are so requisite cannot at all times be secured.

What may be claimed for the treatment, based upon the theory advanced, and pursued as suggested, is, that the death-rate in this terrible disease will be largely diminished, and the amount of suffering immeasurably lessened.

Finally, *the suggestions contained in the foregoing pages can hardly fail to furnish abundant material for discussion;* and if new light shall be thrown upon this obscure disease, by which pain shall be spared or lives prolonged, the effort made in preparing this paper will be amply rewarded.

